# Incidental Finding of Thoracic Aortic Dissection in a Patient Post-Coronary Artery Bypass Graft Surgery

**DOI:** 10.7759/cureus.40443

**Published:** 2023-06-15

**Authors:** Aniekeme S Etuk, Celestine I Odigwe, Sravani Singu, Emmanuel O Amoran, Michael Pursley

**Affiliations:** 1 Internal Medicine, Thomas Hospital, Infirmary Health, Fairhope, USA; 2 Cardiovascular Disease, Northeast Georgia Medical Center Gainsville, Gainsville, USA; 3 Cardiovascular Disease, Thomas Hospital, Infirmary Health, Fairhope, USA

**Keywords:** computed tomography scan, coronary artery by-pass grafting, thoracic aortic aneurysm, thoracic aortic dissection, incidental radiological finding

## Abstract

Thoracic aortic dissection (TAD) is an uncommon but potentially fatal complication of coronary artery bypass graft (CABG). Most patients present to the emergency room with severe chest pain, shortness of breath, or after a syncopal episode. Asymptomatic patients pose a challenge to diagnosis. The authors present a case of an 82-year-old male, who was found to have an incidental finding of a 5-cm ascending aortic aneurysm with an intimal dissection flap four months after CABG. Extensive workup on possible risk factors such as underlying aortic diseases, genetic conditions, and hypertensive crisis proved noncontributory. Aggressive blood pressure control was achieved, and the patient was observed in the intensive care unit before discharge with follow-up. The purpose of this case report is to alert clinicians of TAD after CABG and highlight the importance of developing a protocol for follow-up and monitoring of patients who have undergone CABG, as complications can be asymptomatic. Early and accurate diagnosis of TAD as a complication of CABG is essential to improving survival rates.

## Introduction

Thoracic artery dissection (TAD) is a relatively uncommon but catastrophic condition requiring early diagnosis and management [1]. An aortic dissection is a tear in the intimal layer that creates a false lumen and allows blood to enter and further separate the intimal and media layers [2]. The incidence of TAD is between 2.9 and 4.3 cases per 100,000 persons per year, with a peak age of onset of 55 years and a slight male predominance (male-to-female ratio of 2-3:1) [3]. Uncontrolled hypertension is the leading risk factor for TAD [3]. Others include smoking, aortic aneurysms, inflammatory aortic diseases, inherited connective tissue diseases, trauma, cardiac surgeries, and illicit drug use [3]. A majority of patients with aortic dissection present with sudden tearing chest or back pain. Other common signs and symptoms include dyspnea, loss of consciousness, weak pulses, etc. Patients can also present asymptomatically, especially if they have underlying collagen vascular disease or neurologic impairment [4,5]. Iatrogenic TAD following CABG is uncommon, and there is limited data on the incidence of this phenomenon. Although rare, this complication is fatal [6-8]. We present a unique case of an incidental finding of TAD in a patient post-CABG.

## Case presentation

**Figure 1:**
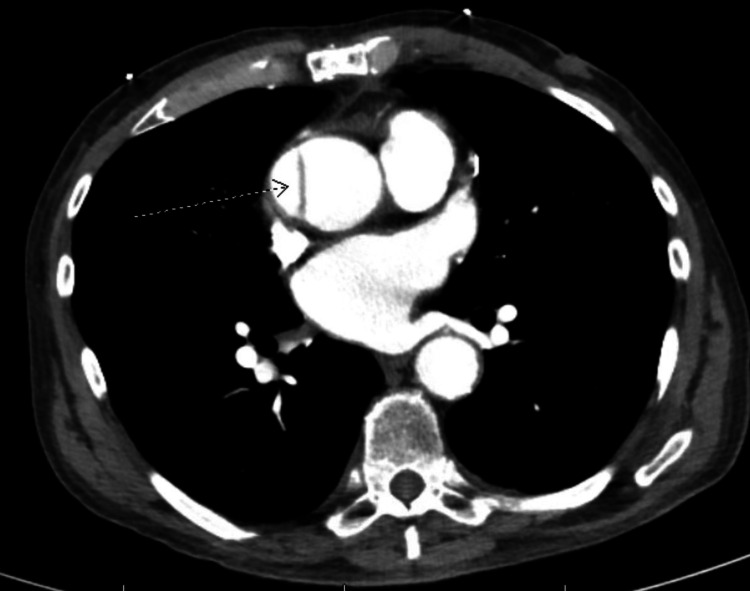
Computed tomography scan showing a 5-cm ascending aortic aneurysm with an intimal dissection flap (see the arrow).

An 82-year-old asymptomatic male presented to the emergency department after an incidental finding of thoracic aortic dissection. He had a medical history of coronary artery disease status post-three-vessel CABG surgery four months before presentation, paroxysmal atrial fibrillation on anticoagulation, gastroesophageal reflux disease, controlled essential hypertension, and peripheral neuropathy. Surgical history only included CABG, and the patient was a former smoker with less than 10-pack-year history. He was undergoing evaluation for a possible malignancy with a computed tomography (CT) scan, which revealed a 5-cm ascending aortic aneurysm with an intimal dissection flap. There was no propagation to the great vessel origins or arch, periaortic hematoma, or evidence of rupture or leak (Figure 1). On presentation, the patient denied chest pain, palpitations, dyspnea, cough, headaches, dizziness, light-headedness, and syncopal events. Blood pressure was 207/112 mmHg, heart rate was 77 beats/minute, and oxygen saturation was 96% on room air. On physical examination, the patient had regular heart sounds without murmurs, rubs, or gallops; clear lungs to auscultation; and a midsternal incision that was clean, dry, and intact.

CBC and comprehensive metabolic panel (CMP) were within normal limits. Cardiac enzymes were trended and negative. Pro brain natriuretic peptide (ProBNP) was elevated at 635 pg/mL (0-450 pg/mL), but this was not significant for a diagnosis of possible heart failure considering his age, as values may be greater than 1,800 pg/mL. The patient had a transthoracic echocardiogram four months before presentation, and it showed a normal ejection fraction with mild asymmetric left ventricular hypertrophy (Video 1).

**Video 1:** Echocardiography showing mild asymmetric left ventricular hypertrophy with normal ejection fraction.

Initially, connective tissue disorders, familial thoracic aortic aneurysm/dissection, preexisting aortic aneurysm, bicuspid aortic valve, aortic coarctation, and inflammatory vasculitis were all considered as possible etiologies of TAD. There was also high suspicion that a hypertensive crisis could have caused dissection; however, the patient reported having blood pressure readings around his baseline of 130/80 mmHg on the day of presentation and for several days before presentation. Further workup for previously mentioned conditions was negative. A diagnosis of thoracic aortic dissection secondary to CABG surgery was made.

Cardiology and cardiothoracic surgery services were consulted, and the decision was made to manage the patient conservatively with blood pressure control in the intensive care unit. Anticoagulation and antiplatelet medications were withheld while monitoring his hematocrit and hemoglobin levels. A repeat CT scan of the chest two days later was stable, and the patient remained asymptomatic. The patient was counseled on the importance of outpatient follow-up and repeat imaging at six months and one year.

## Discussion

TADs are rare but potentially lethal phenomena that can complicate thoracic surgeries [1]. While there have been sketches of data on the occurrence of aortic dissection following thoracic surgeries, concrete data on the exact incidence of TAD post-CABG have been sparse. Iatrogenic aortic dissections occur early, within 30 days, or late, after 30 days postoperatively, and 60% of cases occur during CABG surgery and 17% during aortic valve surgery with or without CABG [9]. TAD site post-CABG varies, relating to the surgical technique [10]. The site of origin of dissection is most commonly the arterial inflow cannula (∼33%). Other common sites are the aortic cross-clamp or partial occlusion clamp (∼29%) [9]. The risk is even more significant in the concomitant weakened aorta, as in collagen vascular diseases, aortitis, calcific vessel wall, or degeneration from advancing age [11].

When the ascending aorta is the site of cannulation, TAD occurs in 0.06% of cases and about 0.5% when the axillary or subclavian arteries are used [9]. The mortality rate is 1% to 2% per hour after symptom onset without surgical intervention and is generally estimated to be 30%. Still, it is more than double if not recognized until the late postoperative period [9,12]. Although chest pain and back pain are the typical presentations, symptoms can be atypical, especially in late TAD, with features such as dyspnea on exertion, dry cough, headache, syncope, anxiety, and fever, mimicking other diagnoses and causing a delay in treatment [13,14].

Our patient was diagnosed with TAD four months after the CABG while on evaluation for a suspicious bony lesion with an oncologist after his CT showed a 5-cm aortic aneurysm with an intimal dissection flap. Interestingly, he had no chest or back pain, and none of the atypical symptoms described in the literature. He showed no features suggestive of neurological complications. As the presentation varies widely, a high suspicion index is necessary. Patients without symptoms pose a significant risk of missed diagnosis and late detection. Perhaps the incidence of TAD post-CABG is much higher than the values described in the literature if we consider possible undiagnosed asymptomatic patients. Unfortunately, while the European System for Cardiac Operative Risk Evaluation (EuroSCORE) can predict the mortality rate after CABG, it does not indicate who is at risk of aortic dissection after CABG. Therefore, there is a need for protocols such as scheduled chest imaging to actively monitor these patients after CABG, as the outcome is graver if dissections are diagnosed much later in the postoperative period either from routine follow-up imaging or when symptoms eventually present [9]. 

## Conclusions

TAD is a rare but potentially fatal complication of CABG that can present several months after the procedure. Therefore, a high index of suspicion is pivotal to timely diagnosis and reducing mortality from this complication. Despite the widely variable presentation of TAD post-CABG, patients can be asymptomatic posing future morbidity and mortality risks. Therefore, there is a need for the development of a systematic approach to monitoring patients postoperatively for early detection and treatment of silent and severe complications.
